# Pulp Regeneration: Current Approaches and Future Challenges

**DOI:** 10.3389/fphys.2016.00058

**Published:** 2016-03-07

**Authors:** Jingwen Yang, Guohua Yuan, Zhi Chen

**Affiliations:** ^1^The State Key Laboratory Breeding Base of Basic Science of Stomatology (Hubei-MOST) and Key Laboratory of Oral Biomedicine Ministry of Education, School and Hospital of Stomatology, Wuhan UniversityWuhan, China; ^2^Department of Pediatric Dentistry, School and Hospital of Stomatology, Wuhan UniversityWuhan, China

**Keywords:** regenerative endodontics, root canal disinfection, cell homing, stem cells, signaling molecules

## Abstract

Regenerative endodontics aims to replace inflamed/necrotic pulp tissues with regenerated pulp-like tissues to revitalize teeth and improve life quality. Pulp revascularization case reports, which showed successful clinical and radiographic outcomes, indicated the possible clinical application of pulp regeneration via cell homing strategy. From a clinical point of view, functional pulp-like tissues should be regenerated with the characterization of vascularization, re-innervation, and dentin deposition with a regulated rate similar to that of normal pulp. Efficient root canal disinfection and proper size of the apical foramen are the two requisite preconditions for pulp regeneration. Progress has been made on pulp regeneration via cell homing strategies. This review focused on the requisite preconditions and cell homing strategies for pulp regeneration. In addition to the traditionally used mechanical preparation and irrigation, antibiotics, irrigation assisted with EndoVac apical negative-pressure system, and ultrasonic and laser irradiation are now being used in root canal disinfection. In addition, pulp-like tissues could be formed with the apical foramen less than 1 mm, although more studies are needed to determine the appropriate size. Moreover, signaling molecules including stromal cell derived factor (SDF-1α), basic Fibroblast Growth Factor (bFGF), Platelet Derived Growth Factor (PDGF), stem cell factor (SCF), and Granulocyte Colony-Stimulating Factor (G-CSF) were used to achieve pulp-like tissue formation via a cell homing strategy. Studies on the cell sources of pulp regeneration might give some indications on the signaling molecular selection. The active recruitment of endogenous cells into root canals to regenerate pulp-like tissues is a novel concept that may offer an unprecedented opportunity for the near-term clinical translation of current biology-based therapies for dental pulp regeneration.

## Introduction

Infected dental pulp is traditionally removed and replaced with inorganic materials (paste and gutta percha) via root canal therapy (RCT). Dental pulp primarily provides nutrition and detects potential pathogens, and the loss of its vitality will increase fragility of the tooth. Therefore, RCT-treated teeth are destined to be devitalized, brittle, and susceptible to postoperative fracture. Thus, an effective treatment strategy is needed to regain vital dental pulp to treat dental pulp diseases. The emergence of modern tissue engineering and regenerative medicine has opened possibilities for regenerative endodontics (Nakashima and Iohara, 2011).

*In vitro* and *in vivo* animal studies have demonstrated great potential for pulp regeneration. Traditionally, three elements, namely (i) stem cells, (ii) scaffolds, and (iii) signaling molecules (e.g., growth factors), were used to achieve pulp regeneration. In the process of pulp regeneration, stem cells were first isolated and manipulated *in vitro*. Then, the cells were loaded onto scaffolds incorporated with signaling molecules and transplanted into the root canal of *ex vivo* tooth slides or *in situ* canine tooth. The formation of pulp-like tissues (connective tissues with blood vessel formation and dentin-like tissue deposition) was observed in many experimental studies (Nakashima and Iohara, 2011; Sun et al., 2014; Yang et al., 2015a,b).

Until now, both stem cell transplantation and cell homing strategies have been applied in pulp regeneration. In the strategy of cell transplantation, stem cells should first be isolated, expanded, seeded into the scaffold, and finally transplanted. Cell homing is aimed to achieve tissue repair/regeneration through recruiting of endogenous cells to injured tissue via signaling molecules. Compared with stem cell transplantation, cell homing strategies do not need to isolate and manipulate stem cells *in vitro*.

Pulp revascularization of immature teeth is a type of cell homing strategy for pulp regeneration in clinical application. It is a two-visit therapeutic approach, which has been proposed in clinical practice over the past decade (Thibodeau and Trope, 2007; Wigler et al., 2013). In this approach, the root canal system is filled with endodontic instrument-induced blood clot after disinfection with a combination of antibiotics. Some case reports have shown the blood flow and sensitiveness to cold or electric stimuli of the tissue are formed in the canal system. This supported the possible application of pulp regeneration strategy without stem cell transplantation for mature teeth in the clinic. However, histological studies have shown that most of the tissues formed in pulp revascularization cases were non-pulp-like tissues comprising cementum, periodontal, and bone-like tissues (Becerra et al., 2014). Moreover, the successful rate of pulp revascularization is low, mainly because of the difficulty in efficient disinfection or induction of blood clot in the canal. Therefore, further studies are needed to facilitate the formation of pulp-like tissues, to increase the success rate of pulp revascularization in immature teeth, and probably to apply this procedure or cell homing strategy in mature teeth.

Possible applications of pulp regeneration strategies continue to evolve, with significant prospects related to the regeneration of functional pulp tissues. The purpose of this review is to provide a detailed overview of present regenerative endodontic approaches aiming to revitalize teeth. From a clinical point of view, we focused on requisite preconditions (including root canal disinfection and enlargement of apical foramen) and cell homing strategies for pulp regeneration.

## Requisite preconditions for pulp regeneration

**Figure 1:**
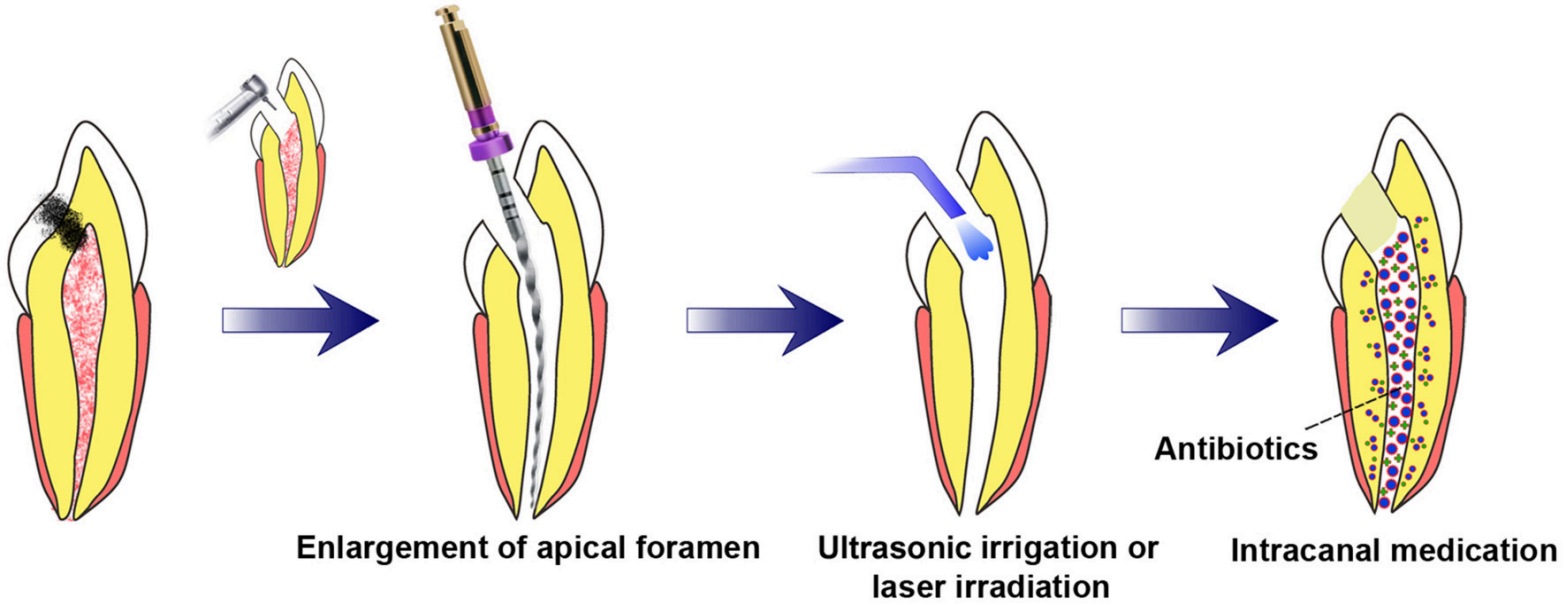
**Requisite preconditions for pulp regeneration (root canal disinfection and enlargement of the apical foramen)**. After mechanical preparation of the root canal combined with irrigation, the apical foramen should be enlarged to a proper size. Root canal disinfection could be achieved through intracanal medication combined with irrigation assisted with EndoVac apical negative-pressure system or ultrasonic and/or laser irradiation.

Two preconditions are requisite to achieve pulp regeneration (Figure 1): (i) efficient root canal disinfection and (ii) proper size of the apical foramen.

During pulp inflammation, diverse oral and food-borne microorganisms invade the pulp space, form biofilm on canal walls, and infiltrate the dentinal tubules (Duggan et al., 2009). To promote regeneration, the pulp space and dentinal walls must be sufficiently disinfected before performing pulp regeneration procedures, and the required degree is possibly higher than that in traditional endodontic therapy (Fouad, 2011). Therefore, efficient root canal disinfection is required for pulp regeneration.

Mechanical preparation, sodium hypochlorite, and calcium hydroxide were the traditionally advocated substances to combat root canal infection in endodontics. However, these methods have been shown to be ineffective, mainly in cases of biofilm-related persistent infections. It was reported that 90% of the bacteria remain positive following irrigation with 10 ml 1.25% sodium hypochlorite (William et al., 2005). Recently, intracanal medication of antibiotics, EndoVac apical negative-pressure system of irrigation, ultrasonic-assisted irrigation, and laser irradiation have been introduced to disinfect root canals.

Clinical studies have proven the potency of triple antibiotic paste (TAP, a combination of metronidazole, ciprofloxacin, and minocycline) as the disinfectant during revascularization (Shivashankar et al., 2012; Vijayaraghavan et al., 2012). It was shown that only 30% of the bacteria remained positive after the application of the TAP for 2 weeks (William et al., 2005). However, there are close relationships between tooth discoloration and minocycline (Lenherr et al., 2012). Therefore, Yassen et al. (2014) suggested the use of double antibiotic paste (DAP, without minocycline) or substitution of minocycline with another antibiotic (clindamyxin, cefaclor, or amoxicillin) for pulp regeneration.

Appropriate use of antibiotics during endodontic regeneration should not only disinfect the root canal but also decrease the adverse effects of antibiotics on transplanted/recruited stem cells. Further, 0.125 mg/ml DAP or TAP has been indicated to exhibit significant antibacterial effects without cytotoxic effects on stem cells (Sabrah et al., 2015). Antibiotic-loaded nanofibrous scaffolds is another method to minimize the adverse effects of highly concentrated antibiotic pastes (Chen, 2010; Bottino et al., 2013; Albuquerque et al., 2015; Kamocki et al., 2015). As the scaffold degrades, antibiotics are released over time. However, the locally sustained release of antibiotics could develop resistant bacterial strains and induce allergic reactions (Lenherr et al., 2012). The application of EndoVac apical negative-pressure system of irrigation, ultrasonic-assisted irrigation, and laser irradiation in root canal disinfection are new methods of root canal disinfection (da Silva et al., 2010; Castelo-Baz et al., 2012; Sahar-Helft et al., 2013; Ghinzelli et al., 2014; Johns et al., 2014; Layton et al., 2015; Neelakantan et al., 2015).

The EndoVac apical negative-pressure system of irrigation, passive ultrasonic irrigation, and laser irradiation have been reported to render similar antibacterial effects to antibiotics (da Silva et al., 2010; Johns et al., 2014; Layton et al., 2015; Neelakantan et al., 2015). *In vitro* studies have shown that continuous ultrasonic-assisted irrigation and NaOCl and EDTA or Ca(OCl)_2_ solutions could aid in chemomechanical preparation and significantly reduce microbial content during root canal treatment (Castelo-Baz et al., 2012). *In vitro* irrigation solutions combined with Er:YAG-laser irradiation are effective in removing Enterococcus faecalis biofilm from root canal walls (Sahar-Helft et al., 2013). In addition, ultrasonic activation could strengthen the elimination of E. faecalis from the root canal (Ghinzelli et al., 2014), although diode and Er:YAG-laser activation have been reported as superior to ultrasonic activation in dentinal tubule disinfection (Neelakantan et al., 2015). Therefore, ultrasonic irrigation and laser irradiation could be used as a substitute of or in combination with antibiotics for tooth disinfection.

Another indispensable precondition for pulp regeneration is the proper size of the apical foramen, especially in mature teeth with closed apex in adults. During tooth root development, the root apex reduces, finally closes, and forms a narrow foramen. This foramen is the only access through which the blood vessels, nerves, and cells inside the dental pulp communicate with surrounding tissues. If the apical foramen is too small in size, it will impact not only the migration of endogenous cells but also the neovascularization and re-innervation during regeneration. Kling et al. (1986) have shown that a minimum of 1.1 mm is necessary to obtain proper revascularization. They found that an apex smaller than 1.0 mm did not allow pulp revascularization in re-implanted permanent incisors. Another study on replantation of avulsed teeth also showed that apices smaller than 1.5 mm have the lowest rate of pulp healing (Andreasen et al., 1995). Size more than 1 mm would remove the majority of the dentin in the apex, which might be too large for teeth and lead to apical trauma or even fracture. Therefore, the apex should be as small as possible, without affecting cell migration, neo-vascularization, and re-innervation. Further, Laureys et al. (2013) reported that 0.32 mm apical foramen did not prevent the ingrowth of new tissue in two-thirds of the pulp chamber 90 days after tooth transplantation. Our previous research has shown that apices approximately 0.8 mm allowed the migration of endogenous cells and blood vessel formation in the root canal of mature dog teeth *in situ* (Yang et al., 2015a). Therefore, it is possible to achieve pulp regeneration through cell homing with the apical foramen less than 1 mm. However, further studies are needed to determine an appropriate size of apical foramen, especially for human teeth. Moreover, the instruments used to enlarge apical foramen should be modified, and a more proper method should be developed to enlarge the apex efficiently and reduce root-fracture risks.

## Essential features of the regenerated pulp tissue

As known, dental pulp is a loosely connective tissue enclosed within rigid dentine walls. There are blood vessels, nerves, and odontoblasts lining the predentine in the dental pulp, which could help supply nutrients, react to infection, and form reactionary dentin, thus maintaining pulp homeostasis (Ricucci et al., 2014). Therefore, the regenerated tissues should be connective tissues that (i) produce new dentin with a controlled rate similar to the normal pulp, (ii) exhibit similar cell density and architecture to the natural pulp, (iii) are vascularized, and (iv) are innervated (Fawzy El-Sayed et al., 2015). Those functional characters of regenerated tissues are more important than the morphological characters are.

Dentinogenesis is an important character of the pulp. However, after being fully developed, the odontoblasts give rise to the secondary dentin at a regulated and low deposition rate (Magloire et al., 2001). Therefore, the regenerated dentin should be formed along the residual dentinal wall with a very low deposition rate similar to that of the normal pulp. No mineralized tissue should be formed in the center of the regenerated tissue. Extensive mineralization in the regenerated pulp will lead to pulp calcification, which will finally result in loss of pulp viability and block the root canal system. This situation will also cause difficulty in re-treatment. Therefore, too much and extensive mineralization should be prohibited.

Vascularization and innervation are the other two characteristics of the pulp. The regenerated blood vessels should have a connection with periapical or bone marrow tissues around the teeth, which could receive a regular blood flow from circulation and supply nutrient to the regenerated tissue and dentin. More importantly, the regenerated tissue should be innervated, so that the teeth are be able to sense hot/cold stimulation and pain during infection (Kökten et al., 2014). Until now, many published studies have examined the dentin deposition and vascularization of the regenerated tissue. However, few studies have been focused on the re-innervation of the tissue. This might be attributed to the limitation of the methods employed for the examination of innervation. Pagella et al. (2014) used the microfluidics co-culture systems to study tooth innervation. They found that microfluidics co-culture systems provided a valuable tool for investigating the innervation in developing or regenerating teeth. Such systems might also be used to analyze the innervation of regenerated dental pulp.

## Cell homing for pulp regeneration

Compared with cell transplantation strategy, the cell homing strategy might be easier to perform in clinic, as there is no need to isolate or manipulate stem cells *in vitro* (Kim et al., 2013; Huang and Garcia-Godoy, 2014; Xiao and Nasu, 2014). In addition to the pulp revascularization in immature teeth, cell homing strategy was introduced and applied in pulp regeneration in a series of studies (Kim K. et al., 2010; Suzuki et al., 2011; Yang et al., 2015a).

Mao's group showed the formation of vascularized connective tissues in the canal with collagen scaffold and a series of molecules (VEGF, PDGF, or bFGF with a basal set of NGF and BMP7). In Mao's study, extracted human canines and incisors were used and treated endodontically without root canal filling materials. After injection of the scaffold combined with molecules into the canal, the teeth were transplanted subcutaneously into mice. Three weeks later, cellularized and vascularized tissues with new dentin formation over the native dentin were observed in some teeth. This is the first study using an ectopic model to demonstrate the formation of pulp-like tissue through the migration, proliferation, and differentiation of host endogenous cells (Kim J. Y. et al., 2010). Pulp-like tissue formation was also found in another study of this group. In Kim's study, anatomically shaped tooth scaffolds mimicking human molar or rat incisor with an embedded mixture of SDF1, BMP7, and neutralized type-1 collagen solution were used to generate tooth-like structures (including pulp) *in vivo* (Kim K. et al., 2010). The scaffold was fabricated by three-dimensional (3D) bioprinting with 200 μm diameter interconnecting microchannels with polycaprolactone (PCL) and HA. This diameter allows for cell migration and proliferation. More importantly, besides formation of periodontal ligament-like tissues and new alveolar bone, vascularized connective tissues were observed in the canal-like space in the model (Kim K. et al., 2010).

Our published data have shown the formation of pulp-like tissues in an *in situ* model using canine mature teeth. In that study, only chemotaxis factor SDF-1α loaded silk fibroin scaffold (pore size 200 μm in diameter) was used. Canine premolars were selected and periapical lesions were first induced. After root canal disinfection, SDF-1α-loaded scaffolds were inserted into the canal following the induction of blood clot. Three months after surgery, formation of pulp-like tissues with neovascularization and dentin formation along the native dentinal wall were observed. The formation of pulp-like tissues in our study might be attributed to the role of SDF-1α in neovascularization and mineralization, as well as its chemotaxis function. Moreover, compared with the blood clot group, no mineralization was observed in the center of the tissues formed in the SDF-1α-loaded scaffold group. However, the innervation of the formed tissue must be further clarified (Yang et al., 2015a).

To initiate the healing potentials of endogenous cells, scaffolds incorporated with different types of signaling molecules should be added. Signal molecules associated with the formation of vessels, nerves, and dentin are used for pulp regeneration. For example, SDF-1α, bFGF and PDGF are molecules for chemotaxis; PDGF and VEGF for vasculogenesis/angiogenesis; NGF for neuronal growth and survival; and BMP-7 for odontoblast differentiation and mineralization (Yang et al., 2015a; Kim J. Y. et al., 2010). Besides those listed, some other signal molecules are indicated to act as a homing factor for pulp regeneration. Stem cell factor (SCF), a powerful chemokine capable of recruiting progenitor cells, has been shown to increase dental pulp cells proliferation and migration. When subcutaneously implanted with collagen sponges, SCF facilitates cell homing, angiogenesis, and tissue remodeling, which indicated suitability of SCF as a potent aid in the regeneration of dental pulp (Pan et al., 2013). G-CSF and bFGF were also employed for pulp regeneration through cell homing (Takeuchi et al., 2015). Both molecules showed similar effect in high migration, proliferation, anti-apoptotic, angiogenic, and neurite outgrowth stimulatory activities *in vitro*. Using an ectopic transplantation model, G-CSF and bFGF led to the regeneration of pulp-like tissues with dentin formation along the dentinal wall.

A major concern in pulp regeneration through cell homing strategy is stem cell sources. Until now, few studies have reported on this issue. The possible cell sources for pulp regeneration through cell homing include dental pulp stem cells (DPSCs), stem cells from apical papilla (SCAP), and bone marrow stem cells (BMSCs), and others.

DPSCs were traditionally used for pulp regeneration through a cell transplantation strategy, and could also be a cell source via cell homing. In infected immature teeth, efficient root canal disinfection could be achieved without mechanical preparation. In this case, some DPSCs with vitality could reside in the root canal system. Studies of Mitsiadis's group have shown that in seriously injured or carious teeth, stem cells residing in the dental pulp are responsible for the repair and regeneration of the damaged dental tissues (Mitsiadis et al., 2011; Mitsiadis and Woloszyk, 2015). In addition, DPSCs from inflamed dental pulp (DPSCs-IPs) have been identified and shown to exhibit similar mesenchymal stem cell properties to those from normal pulp (DPSCs-NPs) (Alongi et al., 2010). DPSCs-IPs could form pulp/dentin complexes when transplanted in immune-compromised mice. Moreover, they exhibit considerably lower osteo/dentinogenic potential than DPSCs-NPs do on the basis of mineral deposition in cultures. However, it is very difficult to get DPSCs-IPs in mature teeth after mechanical preparation and root canal disinfection. Therefore, DPSCs residual in the canal might contribute to pulp regeneration in immature teeth.

SCAP might be another possible cell source for pulp regeneration. Apical papilla is apical to the epithelial diaphragm of the immature teeth, and it has the collateral circulation. Moreover, there is an apical cell-rich zone lying between the apical papilla and the pulp (Saito et al., 2015). Therefore, the apical papilla is able to survive during the process of pulp necrosis. SCAPs were first identified by Sonoyama et al. Although, shown to have somewhat different characteristics compared with DPSCs, SCAPs have been highly proliferative in cultures and have a strong odontogenic differentiation capacity (Sonoyama et al., 2008). The *in vivo* implantation of SCAP with a scaffold allowed the formation of pulp-like tissues in the root canal (Huang et al., 2010). Na et al. (2013) have also reported the formation of heterotopic dental pulp/dentine complex in empty root canals using SCAP-cell sheet-derived pellet without a scaffold. In addition, SCAPs were shown to be chemoattracted via the SDF-1a/CXCR4 axis and were used as an exogenous cell source to regenerate pulp- and dentin-like tissues in root canal spaces in animal models (Liu et al., 2015). Therefore, SCAPs might be a cell source for pulp regeneration in immature teeth.

Cells homed from bone marrow could be another source for pulp regeneration. Bone marrow–derived progenitor cells have been reported to communicate with dental pulp in mature teeth and become tissue-specific mesenchymal progenitor cells to maintain pulp homeostasis (Zhou et al., 2011). This result indicated that bone marrow-derived cells were capable of migrating into the root canal and participating in pulp formation. Moreover, transplantations of CD31^−^ SP BMSCs or G-CSF-mobilized BMSCs could induce pulp-like tissue formation in the pulpectomized root canal of dogs. In their studies, pulp-like connective tissues with vascularization, innervation, and collagenous matrix containing dentin sialophosphoprotein positive cells were formed (Ishizaka et al., 2013; Murakami et al., 2015).

In addition to the aforementioned possible cell sources, cells of periodontal ligament might be recruited to the root canal system by the chemokines. The periodontal ligament is a fibrous connective tissue structure that joins the cementum covering the root to the alveolar bone. Undifferentiated cells in the periodontal ligament could differentiate into the specialized cells that form bone (osteoblasts) and cementum (cementoblasts). When periodontal ligament stem cells are transplanted with HA/TCP into immune-compromised mice, cementum-like structures associated with periodontal ligament-like connective tissue are generated (Shi et al., 2005). Therefore, the presence of cementum and bone-like tissues in the root canals of some pulp revascularization cases might be attributed to the recruitment of periodontal ligament cells (Wang et al., 2010; Becerra et al., 2014). Thus, the recruitment of periodontal ligament cells should be prohibited to achieve the formation of pulp-like tissues.

In summary, although vascularized pulp-like tissues have been formed in some experimental and clinical studies, little has been known on the function of the regenerated tissue. Further, studies are needed to achieve functional pulp regeneration. Studies on the cell sources of pulp regeneration via cell homing strategy might reveal more information on the signaling molecular selection. The proper signaling molecules for pulp regeneration should facilitate the recruitment of stem cells with the vasculogenic and neurogenic differentiation potential, while inhibiting cells with osteogenic or cementogenic potential (e.g., periodontal ligament cells).

## Future prospect and challenges in pulp regeneration through cell homing

**Figure 2:**
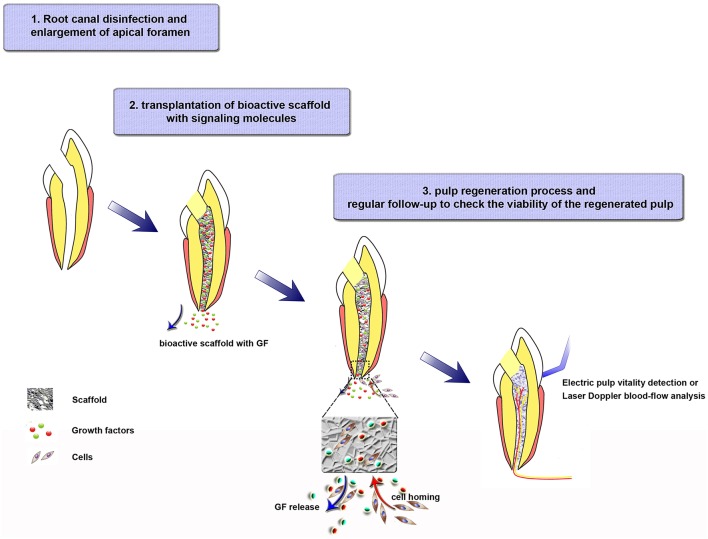
**Tissue-engineering strategies for regenerative endodontics through a cell homing strategy**. Step 1. Root canal disinfection and enlargement of apical foramen. Step 2. Bioactive scaffold transplantation and tooth restoration. Load bioactive scaffold in the canal after cleaning the antibiotics effectively. The growth factors, which are released from the scaffold to the surrounding tissues, will recruit cells into the scaffold. Step 3. Pulp-formation process. Recruited cells migrate, proliferate, differentiate in the scaffold, and participate in the formation of blood vessels, nerves, and regulated dentin deposition. During this step, regular follow-up to check the viability of the regenerated pulp are needed.

Cell transplantation and cell homing are both scientifically meritorious approaches. However, the active recruitment of endogenous stem/progenitor cells into root canals to regenerate pulp tissues is a novel concept that may offer an unprecedented opportunity for the near-term clinical translation of current biology-based therapies for dental pulp regeneration. Three clinical procedures should be performed if cell homing is applied in endodontic treatment (Figure 2): (i) root canal disinfection and apical foramen enlargement; (ii) transplantation of bioactive scaffold with signaling molecules, and tooth restoration; (iii) regular follow-up to check the viability (neovascularization and re-innervation) of the regenerated pulp. Further, studies are needed to achieve clinical translation of pulp regeneration via cell homing strategy. First, for root canal disinfection, intracanal medication, irrigation assisted with EndoVac apical negative-pressure system, or ultrasonic and laser irradiation can be used, but the antibacterial efficiency, effects on cell viability, and dentin mechanical strength of each method or a combination of two or more of them must be further clarified. Meanwhile, the appropriate size of apical foramen should be determined. The size should be as small as possible without affecting cell migration and formation of blood vessels and nerves during pulp regeneration. Second, an appropriate combination of scaffold and growth factors must be selected. The signal molecules should recruit stem cells with the vasculogenic and neurogenic differentiation potential, but inhibit cells with osteogenic or cementogenic potential. In addition, the scaffold should be easily handled in clinical practice. Finally, long-term follow-up experiments will be necessary before the clinic application of cell homing strategy in humans.

## Funding

This work was supported by grants from the National Natural Science Foundation of China (81420108011, 31500788); the Fundamental Research Fund for the Central Universities (410500114).

### Conflict of interest statement

The authors declare that the research was conducted in the absence of any commercial or financial relationships that could be construed as a potential conflict of interest.
